# Entwicklung und Evaluation eines Deep-Learning-Algorithmus für die Worterkennung aus Lippenbewegungen für die deutsche Sprache

**DOI:** 10.1007/s00106-021-01143-9

**Published:** 2022-01-13

**Authors:** Dinh Nam Pham, Torsten Rahne

**Affiliations:** 1 Universitätsklinik und Poliklinik für Hals‑, Nasen‑, Ohrenheilkunde, Kopf- und Halschirurgie, Universitätsklinikum Halle (Saale), Martin-Luther-Universität Halle-Wittenberg, Ernst-Grube-Str. 40, 06120 Halle (Saale), Deutschland; 2 Hochschule Anhalt, Köthen, Deutschland

**Keywords:** Visuelle Spracherkennung, Lippenlesen, Künstliche Intelligenz (KI), Merkmalsextraktion, Neuronale Netze, Visual speech recognition, Lipreading, Artificial Intelligence (AI), Feature extraction, Neural networks

## Abstract

**Hintergrund:**

Zahlreiche Menschen profitieren beim Lippenlesen von den zusätzlichen visuellen Informationen aus den Lippenbewegungen des Sprechenden, was jedoch sehr fehleranfällig ist. Algorithmen zum Lippenlesen mit auf künstlichen neuronalen Netzwerken basierender künstlicher Intelligenz verbessern die Worterkennung signifikant, stehen jedoch nicht für die deutsche Sprache zur Verfügung.

**Material und Methoden:**

Es wurden 1806 Videos mit jeweils nur einer deutsch sprechenden Person selektiert, in Wortsegmente unterteilt und mit einer Spracherkennungssoftware Wortklassen zugeordnet. In 38.391 Videosegmenten mit 32 Sprechenden wurden 18 mehrsilbige, visuell voneinander unterscheidbare Wörter zum Trainieren und Validieren eines neuronalen Netzwerks verwendet. Die Modelle 3D Convolutional Neural Network, Gated Recurrent Units und die Kombination beider Modelle (GRUConv) wurden ebenso verglichen wie unterschiedliche Bildausschnitte und Farbräume der Videos. Die Korrektklassifikationsrate wurde jeweils innerhalb von 5000 Trainingsepochen ermittelt.

**Ergebnisse:**

Der Vergleich der Farbräume ergab keine relevant unterschiedlichen Korrektklassifikationsraten im Bereich von 69 % bis 72 %. Bei Zuschneidung auf die Lippen wurde mit 70 % eine deutlich höhere Korrektklassifikationsrate als bei Zuschnitt auf das gesamte Sprechergesicht (34 %) erreicht. Mit dem GRUConv-Modell betrugen die maximalen Korrektklassifikationsraten 87 % bei bekannten Sprechenden und 63 % in der Validierung mit unbekannten Sprechenden.

**Schlussfolgerung:**

Das erstmals für die deutsche Sprache entwickelte neuronale Netzwerk zum Lippenlesen zeigt eine sehr große, mit englischsprachigen Algorithmen vergleichbare Genauigkeit. Es funktioniert auch mit unbekannten Sprechenden und kann mit mehr Wortklassen generalisiert werden.

## Abstract (en)

**Background:**

When reading lips, many people benefit from additional visual information from the lip movements of the speaker, which is, however, very error prone. Algorithms for lip reading with artificial intelligence based on artificial neural networks significantly improve word recognition but are not available for the German language.

**Materials and methods:**

A total of 1806 videoclips with only one German-speaking person each were selected, split into word segments, and assigned to word classes using speech-recognition software. In 38,391 video segments with 32 speakers, 18 polysyllabic, visually distinguishable words were used to train and validate a neural network. The 3D Convolutional Neural Network and Gated Recurrent Units models and a combination of both models (GRUConv) were compared, as were different image sections and color spaces of the videos. The accuracy was determined in 5000 training epochs.

**Results:**

Comparison of the color spaces did not reveal any relevant different correct classification rates in the range from 69% to 72%. With a cut to the lips, a significantly higher accuracy of 70% was achieved than when cut to the entire speaker’s face (34%). With the GRUConv model, the maximum accuracies were 87% with known speakers and 63% in the validation with unknown speakers.

**Conclusion:**

The neural network for lip reading, which was first developed for the German language, shows a very high level of accuracy, comparable to English-language algorithms. It works with unknown speakers as well and can be generalized with more word classes.

## Hintergrund und Fragestellung

Zahlreiche Menschen im deutschsprachigen Raum leiden unter einer Hörstörung [10]. Je größer und länger der Hörverlust besteht, desto mehr profitieren solche Menschen auch von den zusätzlichen Informationen aus den Lippenbewegungen des Sprechenden [1, 28]. Aber auch Normalhörende können hierdurch in anspruchsvollen, vor allem geräuschbelasteten akustischen Situation besser verstehen [26, 38]. Dieses sogenannte Lippenlesen ist jedoch fehleranfällig. So sind in der deutschen Sprache nur ungefähr 15 % der Laute am Mundbild erkennbar. Bei Einbettung in den Kontext erhöht sich die Trefferquote einer Schätzung zufolge auf 50 % [20]. Für englischsprachige Sprecher sind je nach experimentellem Design der Studie Trefferquoten von 40–70 % bekannt [16, 34].

In den letzten Jahren wurde erfolgreich versucht, Lippenlesen durch Algorithmen mit auf künstlichen neuronalen Netzwerken basierender künstlicher Intelligenz (KI) zu verbessern [15]. Für die englische Sprache wird KI z. B. mit Ansätzen von LipNet [2], dem ersten End-to-end-Deep-Learning-Modell für das Lippenlesen auf Satzebene durch Vorhersagen von Zeichensequenzen mittels Connectionist Temporal Classification Loss, sowie von Google Deep Mind [29], welches eine Pipeline zur Generierung eines groß angelegten Datensatzes aus Videoquellen einführt und, im Gegensatz zu LipNet, Phoneme statt Zeichen vorhersagt, verwendet. Derzeit bestehen jedoch keine Anwendungen für die deutsche Sprache.

Menschen mit plötzlich erworbenem, das Sprachverstehen signifikant reduzierenden Hörverlust haben oft Mühe, Sprechende in ruhigen, vor allem aber auch in geräuschvollen Umgebungen zu verstehen. Die Fähigkeit, aus Lippenbewegungen Sprachinformationen ablesen zu können, muss in den meisten Fällen erst erlernt werden, um im Alltag relevant nützlich zu werden. Im umgekehrten Fall kann aber auch die Artikulationsfähigkeit von Sprache deutlich reduziert sein. Nach Kehlkopfoperation und Anlage eine Tracheostomas ist die akustische Sprachproduktion meist nicht mehr möglich, wohingegen die Lippenbewegungen nahezu unverändert sprachkorreliert erfolgen können. Solche Menschen verstehen zu können, ist für die Kommunikationspartner oder Pflegende sehr wichtig, jedoch oft nur schwer möglich. Hier wären technisch niederschwellige Algorithmen zur Spracherkennung aus Lippenbewegungen sehr hilfreich.

Aufgaben wie das Lippenlesen stellen Herausforderungen dar, wozu bislang keine Algorithmen bekannt sind, welche diese vollständig und optimal lösen. Auch Menschen besitzen nicht die Fähigkeit, in jedem Fall die Lippenbewegung eindeutig in Sprache zu übersetzen. Jedoch ist beim Lippenlesen ein großer Lerneffekt zu beobachten [16, 34]. Um auch durch Computer möglichst hohe Genauigkeiten beim Lippenlesen zu erreichen, wurden Deep-Learning-Methoden verwendet, welche auf tiefen künstlichen neuronalen Netzwerken basieren. Dabei werden mithilfe von Trainingsbeispielen unter anderem die Gewichtungen zwischen den Neuronen verändert. Aus dem Training folgt schließlich eine Generalisierungsfähigkeit, um erlerntes Wissen mit einer deutlich reduzierten Fehleranfälligkeit auf neue Beispiele anzuwenden. Neuronale Netzwerke können als universelle Approximatoren betrachtet werden, welche mit genügend Neuronen in der Theorie *n*-dimensionale Funktionen darstellen können [13]. Unter der Annahme, dass Lippenlesen eine Aufgabe ist, bei dem erkennbare Zusammenhänge beziehungsweise Muster auftreten, ist der Erfolg künstlicher neuronaler Netzwerke beim Lernen von Lippenlesen zu erklären.

Als essenzieller Bestandteil der meisten Deep-Learning-Modelle dient das mehrschichtige Perzeptronen-Netzwerk (Multilayer Perceptron [MLP]) als ein universeller Approximator von Funktionen [8], sodass also unter anderem Regressionsanalysen und Klassifikationen ermöglicht werden. Neuronen als kleinste Recheneinheit in solchen Netzwerken besitzen *n* Eingänge, die auch von anderen Perzeptronen stammen können. Jeder Eingang verfügt jeweils über ein Gewicht *w*_i_. Die Ausgabe des Perzeptrons ist die um ein gewichtetes Bias ergänzte Summe der gewichteten Eingänge in einer Aktivierungsfunktion. Für eine höhere Aussagekraft werden mehrere Perzeptronen zu einem mehrschichtigen Perzeptronen-Netzwerk zusammengefasst. Jedes Perzeptron einer Schicht erhält die Ausgaben des vorherigen Layers als Eingang. Zur Approximation nichtlinearer komplexer Zusammenhänge sind im neuronalen Netzwerk nichtlineare Aktivierungsfunktionen notwendig. Diese sorgen dafür, dass Modelle komplexere Zusammenhänge zwischen Eingaben und Ausgaben herstellen, was für Problemstellungen wie das Lippenlesen essenziell ist. In dieser Arbeit ist die am weitesten verbreitete Rectified Linear Unit(ReLU)-Funktion [36] in den versteckten Schichten und die Softmax-Funktion [3] in den Ausgabeschichten relevant. Die ReLU-Funktion gibt das Argument aus, sofern dieses größer als Null ist. Ansonsten wird 0 als Funktionswert ausgegeben. Die Softmax-Funktion ist eine normalisierte Exponentialfunktion und berechnet die Wahrscheinlichkeitsverteilung eines Vektors mit reellen Komponenten. Dies wird bei der Ausgabeschicht für die Multiklassen-Klassifizierung genutzt. Jede Komponente steht für eine Klasse, sodass die wahrscheinlichste Klasse klassifiziert wird. Insgesamt kann ein MLP mithilfe von genügend Neuronen und Schichten und nichtlinearen Aktivierungsfunktionen beliebige *n*-dimensionale Funktionen approximieren.

Neben den mehrschichtigen Perzeptronen-Netzwerken werden als weitere Klasse neuronaler Netze 2‑D-Convolutional-Layer betrachtet, die eine festgelegte Anzahl an Filtern (Kernels) besitzen [23]. Diese sind Matrizen mit reellen Elementen und haben eine feste Größe. Die Kernel-Size und die Anzahl an Filtern sind Parameter, welche beim Entwurf eines Convolutional Neural Network (CNN) zu beachten sind. Beim Convolutional-Layer werden Filter schrittweise über die Input-Matrix geschoben. Dabei wird das Kernel zeilenweise von links nach rechts mit festgelegter Schrittweite (Stride) bewegt. Jedes Mal, bevor das Kernel gleitet, werden die Komponenten der Input-Matrix mit ihren zugehörigen Gewichtungen im Kernel multipliziert, und schließlich wird die Summe gebildet. Diese Summe wird zu einem Element der ausgegebenen Feature-Map-Matrix. Das Aktualisieren der Gewichtungen in den Filtern sorgt für das Lernen der Mustererkennung bzw. Extraktion der Features. Die Anzahl an Kernels bestimmt, wie tief die entstandene Feature-Map ist. Während 2‑D Convolutional Neural Networks (CNN oder ConvNet) ursprünglich zum Lösen von Computer-Vision-Problemen, wie beispielsweise Handschrifterkennung, entwickelt wurden [21], erreichen dreidimensionale CNN bei 3‑D-Bildern höhere Genauigkeiten [22]. Videos können als vierdimensionale Arrays mit Dimensionen für Kanäle, Tiefe, Höhe und Breite dargestellt werden und sind somit für 3‑D-CNN geeignet. Anders als MLP, welche ausschließlich Vektoren als Eingabe verarbeiten können, sind CNN in der Lage, Input in Form von Matrizen, also auch Bilder, zu verwenden. Mit vielen Kernels und mehreren aufeinanderfolgenden Schichten können komplexere Muster erkannt werden. Im Fall von Videos können 3‑D-CNN nicht nur die bildlichen, sondern möglicherweise auch erste zeitliche Informationen extrahieren.

Im Gegensatz zu den genannten Feedforward-Schichten [35], bei dem die Ausgaben stets an die nächste Schicht weitergeleitet wurden, eignen sich für die Verarbeitung sequenzieller Daten vor allem rekurrente neuronale Netze, bei denen Rückkopplungen durchgeführt werden [7, 13]. Die Gated Recurrent Units (GRU) [6] eignen sich zur Erfassung der zeitlichen Informationen von Videos mit relativ geringem Rechenaufwand. Das Reset Gate wird als Vektor bestimmt, bei dem vergangene Informationen gelöscht, also vergessen, werden sollen. Das Update Gate bestimmt, welche Informationen der versteckten Zustände vergangener Zeitschritte behalten werden sollen. Durch Eingabe von Trainingsbeispielen wird mit der Zeit gelernt, zeitliche Informationen zu extrahieren, um bestmögliche Vorhersagen zu treffen. Anders als CNN, können GRU und MLP aber ausschließlich Vektoren verarbeiten. Sollen die Ausgaben von CNN, was drei- oder vierdimensionale Arrays sind, also in diesen Schichten übertragen werden, so müssen diese in Vektoren bzw. eindimensionale Arrays transformiert werden (Flattening).

Automatische Algorithmen zur Erkennung von Lippenlesen benötigen zum Training eine große Datenbasis. Diese steht für den deutschen Sprachraum nicht zur Verfügung. Die Größe, Qualität und Relevanz der Datenbasis bestimmt den Erfolg des Modells maßgeblich [15]. Die Spracherkennungsrate der automatischen Algorithmen zur Spracherkennung aus Lippenbewegungen variiert stark und hängt vom verwendeten Algorithmus und der Datenbasis ab. Mit dem von Chung et al. [7] vorgestellten, auf CNN und RNN basierenden neuronalen Netz konnten 47 % der englischen Sätze einer Datenbasis mit 10.000 Items korrekt erkannt werden. Eine sehr große Datenbasis für die chinesische Sprache wurde von Yang et al. [37] vorgestellt. Einen Überblick gibt Hao [15] und listet z. B. Erkennungsraten für Wörter von 76 % [7] bis 98 % [33] für die englische Sprache auf. Für die deutsche Sprache wurden bisher keine Algorithmen mit großen deutschsprachigen Datensätzen entwickelt. Auf neuronalen Netzen basierende Algorithmen zur Erkennung von Lippenlesen scheinen sprachübergreifend wirksam zu sein. Die phonologische Interferenz zwischen den eng verwandten westgermanischen Sprachen Deutsch und Englisch lässt vermuten, dass sich solche Algorithmen auch auf die deutsche Sprache übertragen lassen.

Ziel der Arbeit ist es, durch die Konzeption und Implementierung eines geeigneten neuronalen Netzwerks das automatisierte Lippenlesen auf Wortebene für Deutschsprechende im Sinne einer visuellen Spracherkennung zu entwickeln. Hierzu wird ein Datensatz erstellt, der zum Training und Evaluierung der Deep-Learning-Modelle dient. Dabei wird die Genauigkeit von sowohl im Training bekannten als auch unbekannten Sprechern untersucht. Um eine möglichst hohe Genauigkeit zu erzielen, werden verschiedene Netzwerkarchitekturen und Methoden der Vorverarbeitung der Eingabedaten verglichen. Es wird eine Prozesskette entwickelt, die Eingabedaten von Sprechern ohne Ton verarbeitet und durch das Modell klassifiziert.

## Material und Methoden

### Datensatz

Zunächst wurden 1806 Videos, bei denen jeweils nur eine deutsch sprechende Person klar identifizierbar ist, auf der Videoplattform YouTube (YouTube LCC, San Bruno, USA) selektiert und zur weiteren Bearbeitung lokal gespeichert. Insbesondere Videos, die zum Deutschlernen gedacht sind, wurden ausgewählt. Alle Originalvideos besaßen eine FPS von 25 und eine Auflösung von 1280 × 720 Pixeln (in Einzelfällen auch 640 × 360 Pixel). Mittels der Open-Source-Spracherkennungssoftware Vosk API [30] wurden die in einer Sequenz fließender Sprache mit entsprechender Koartikulation ausgesprochenen Wörter identifiziert und die dazugehörigen Zeitstempel ermittelt. Die automatische Worterkennung wurde manuell verifiziert. Für den Datensatz werden die folgenden 18 mehrsilbigen, visuell voneinander unterscheidbaren Wörter als Wortklassen verwendet: kommentare – fragen – prüfung – deutschland – können – sprechen – wirklich – eigentlich – wissen – natürlich – video – bedeutet – beispiel – schreiben – menschen – einfach – wichtig – wörter.

Der vollständige Datensatz besteht somit aus 38.391 Videos mit 32 Sprecher:innen, wobei in jedem Video eine Sprecher:in eines der 18 Wörter ausspricht. Dieser Datensatz wurde in drei Teildatensätze (A, B, C) ohne Überlappung geteilt. Jeder dieser Teildatensätze besteht aus Videos unterschiedlicher Sprechender. Datensatz A wurde zudem in einen Trainings- und Validierungssatz im Verhältnis 8:2 untergliedert. Datensatz B wurde in Trainings‑, Validierungs- und Testdatensätze im Verhältnis 8:1:1 unterteilt (Tab. 1).

**Table 1:** 

Datensatz		Originalvideos	Sprechende	Videos im Datensatz
A	Gesamt	250	6	3727
	Training			2973
	Validierung			754
B	Gesamt	1400	22	30.684
	Training			24.553
	Validierung			3060
C	Test	156	4	3950
	Gesamt			3950

In beiden Datensätzen wurde bei der Unterteilung das Verhältnis der Anzahl an Videos in den Klassen übernommen, sodass sowohl im Trainings- als auch im Validierungssatz die Klasse mit den meisten oder wenigsten Videos die gleiche ist. Damit wird verhindert, dass die Mehrzahl an Videos einer Klasse sich im Validierungssatz befindet und nicht genügend Beispiele dieser Klasse im Trainingssatz zum Lernen vorhanden sind. Jedes Video in den Validierungs- und Testsätzen der Datasets wurde manuell überprüft, und Videos mit falschen Wörtern wurden entfernt.

### Videobearbeitung

**Figure 1:**
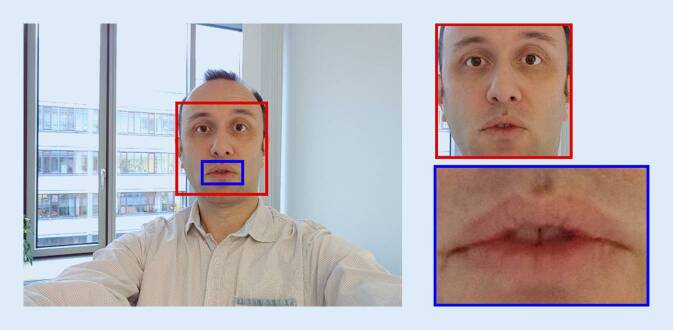

Alle Videos wurden auf eine einheitliche Länge von 28 Frames gebracht. Überzählige Frames wurden entfernt und Videos mit weniger Frames durch wiederholtes Verwenden des letzten Frames auf diese Länge gebracht. Die so bearbeiteten Videos wurden als 4‑dimensionales (Farbkanal, Frame, Höhe, Breite), auf 0–1 normiertes Array gespeichert. In jedem Frame wird zum einen der Gesichtsbereich, zum anderen der Mund automatisch identifiziert und jedes Video entsprechend beschnitten. Die erkannten Gesichtsbereiche wurden auf 90 × 90 Pixel, die Mundbereiche auf 150 × 100 Pixel durch lineare Interpolation up- oder downgesampled. Die Abb. 1 zeigt ein Beispiel der verwendeten Bildausschnitte. Weil unterschiedliche Farbräume zu unterschiedlichen Genauigkeiten in der Bildklassifizierung führen können [14], wurden die Videos jeweils in die Farbräume RGB, Graustufen, HSV, LAB, XYZ und YcbCr konvertiert.

### Modelle und Training

Zum Trainieren des neuronalen Netzwerks wurden die Modelle 3D Convolutional Neural Network (Conv3D), Gated Recurrent Units (GRU) und GRUConv, eine Kombination der ersten beiden Modelle, verwendet.

Als erste Schicht des Modells Conv3D wird eine Batch-Normalisierung [17] der Eingabedaten durchgeführt, sodass der Mittelwert 0 die Standardabweichung 1 beträgt. Dies soll die Performance des Modells verbessern und zu einem stabileren Trainingsprozess führen. Danach folgt eine 3‑D-CNN-Schicht, MaxPooling sowie eine erneute Batch-Normalisierung. Alle drei Schichtarten wurden in dieser Reihenfolge dreimal wiederholt. Alle drei CNN- sowie MaxPooling-Schichten werden mit einem Padding von (1, 2, 2) verwendet. Während alle Convolutional-Layers eine Kernel-Size von (3, 5, 5) besitzen, haben die MaxPooling-Schichten die Kernelgröße (1, 2, 2). Das erste 3‑D-CNN ist mit einem Stride von (1, 2, 2) konfiguriert, die beiden weiteren mit (1, 1, 1). Die Anzahl der Kernels der drei CNN-Schichten betragen 8, 16 und 32. Nach einem abschließenden Flattening und MLP, welches 500 Neuronen in der versteckten Schicht beinhaltet, gibt das Modell einen Vektor mit 18 Komponenten aus. Mit der Softmax-Funktion wurden die Wahrscheinlichkeiten für die Wortklassen bestimmt. Deren Maximum ergab das als Ausgabe definierte Wort. Als Aktivierungsfunktion wurde bei den 3‑D-CNN sowie dem MLP die ReLU-Funktion genutzt. Nach jedem MaxPooling sowie beim MLP wurde ein Dropout [31] von 0,5 angewandt. Dies bedeutet, dass zufällige 50 % der Neuronen der Schicht beim Training ignoriert werden.

**Figure 2:**
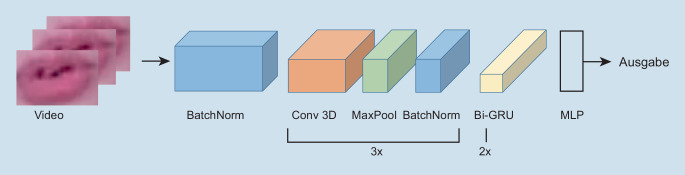

Pro Lernbeispiel und Iteration im Training wurden immer wieder zufällige Neuronen ausgewählt. Damit wurde die Gefahr von Überanpassung verringert, bei der ein Modell hohe Genauigkeiten beim Trainingssatz erzielt, aber bei ungesehenen Testdaten mit fortschreitendem Training niedrigere Genauigkeiten erzielt. Die Abb. 2 zeigt die Reihenfolge der im Modell verwendeten Schichten.

Im GRU-Modell wurde zunächst pro Frame ein Flattening durchgeführt. Bei 3 Kanälen und einer Bildauflösung von 150 × 100 Pixeln ergibt dies einen Vektor mit 45.000 Elementen je Frame mit insgesamt 28 Frames. Diese Daten mit einer Dimensionalität von 28 × 45.000 wurden als Eingabe für zwei bidirektionale GRU-Schichten verwendet, wobei der Hidden-State-Vektor der ersten GRU-Schicht eine Dimensionalität von 1000 und die der zweiten Schicht eine Dimensionalität von 512 besitzt. Ein bidirektionales GRU-Layer besteht aus zwei GRU-Schichten, sodass die Eingabesequenz in einer der Schichten unverändert eingespeist wird und in die andere in umgekehrter Reihenfolge. Die versteckten Zustände dieser beiden einzelnen GRU-Schichten werden konkateniert und bilden anschließend den Hidden-State-Vektor der bidirektionalen GRU-Schicht, um das Lernen von Sequenzen auch in umgekehrter Reihenfolge zu ermöglichen. Die Ausgabe des ersten birektionalen GRU-Layers wird als Eingabe für die zweite GRU-Schicht verwendet. Die Ausgabe des zweiten bidirektionalen GRU wurde schließlich in ein MLP eingespeist, welches die gleiche Konfiguration wie das MLP des Conv3D-Modells besitzt.

Im GRUConv-Modell wurden zwischen Schichten des Conv3D-Modells und dem MLP die zwei GRU-Schichten des GRU-Modells eingefügt. Während dreidimensionale CNN räumliche Informationen extrahieren, sind GRU für zeitliche Informationen gut geeignet.

**Figure 3:**
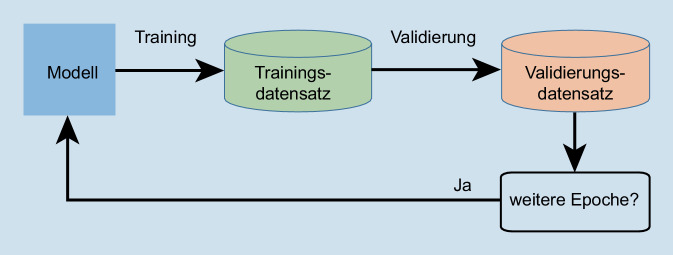

Beim Training jedes der Modelle wurden mehrere Epochen iteriert. In jeder Epoche wurde das Modell mit jedem Video im Trainingssatz mit einer Lernrate von 0,000001 trainiert, sodass die lernbaren Parameter des neuronalen Netzwerks aktualisiert wurden. Als Optimierungsalgorithmus wurde eine adaptive Momentschätzung (ADAM, [19]) und als Lossfunktion die Kreuzentropie verwendet. Anschließend wurde jedes Video im Validierungssatz klassifiziert. Die Anzahl an richtig klassifizierten Videos geteilt durch die Gesamtanzahl der Videos im Validierungssatz ergab die Genauigkeit der Epoche. Die erlernten Parameter in der Epoche mit der besten Validierungsgenauigkeit wurden für jedes Modell bestimmt und für die spätere Evaluierung verwendet. Die Batchgröße für den Trainingssatz betrug 128 und für den Validierungssatz 32. In Abb. 3 wird die beschriebene Trainingsstrategie schematisch dargestellt.

### Implementierung

Die Erstellung der Datensätze, die Prozesskette, die Modelle und der Trainingsprozess wurden in der Programmiersprache Python umgesetzt. Zunächst wurden für den Datensatz die YouTube-Links manuell gesammelt. Mithilfe von youtube-dl [12] wurden die Videos anschließend heruntergeladen. Für die Spracherkennung der Videos wurde das Python-Modul der Vosk API [30] verwendet, welches ein fertiges Sprachmodell für die deutsche Sprache beinhaltet. Da diese wiederum Audiodateien als Eingabe erwartet, wurde die Open-Source-Software FFmpeg [32] zur Extrahierung des Audiomaterials (WAV-Dateien) aus den Videos (AVI-Dateien) verwendet.

Das Python-Modul MoviePy [5] ermöglichte anschließend die Extrahierung der einzelnen Videosequenzen nach den erkannten Videozeiten der Wörter. Danach wurden die Videos zum Zuschneiden der Sprecher mithilfe der Programmierbibliothek OpenCV [4] gelesen und durch das Python-Modul Face Recognition [11] auf die Gesichter zugeschnitten. Das Extrahieren der Lippenbildausschnitte selbst wurde mit den Toolkits Dlib [18] und imutils [27], insbesondere unter Verwendung ihrer 68-Punkt-Gesichtsmerkmalsdetektoren, umgesetzt. Die Aufteilung des Datensatzes in Trainings‑, Validierungs- oder auch Testsatz sowie das Konvertieren der Videos in Farbräume erfolgte über die Programmierbibliothek Scikit-learn [25].

Die KI-Modelle und deren Training wurden mit der Deep-Learning-Bibliothek PyTorch [24] umgesetzt. Als Hardware wurden 4‑Tesla-V100-SXM2-Grafikprozessoren (Fa. Nvidia Corp., Santa Clara, USA) mit jeweils 32 GB Speicher verwendet. Dabei wurde Modell-Parallelismus angewandt, das heißt, die Schichten der neuronalen Netzwerke wurden auf unterschiedliche GPU verteilt.

Die Genauigkeit des Lippenlesens wurde innerhalb von 5000 Trainingsepochen mit dem Conv3D-Modell ermittelt. Dabei wurden die zwei Teildatensätze des Datensatzes A (Tab. 1) zum Trainieren und Validieren verwendet und im RGB-Farbraum das Zuschneiden auf das Gesicht mit dem Zuschneiden auf den Mundbereich hinsichtlich der Validierungsgenauigkeit verglichen.

Der Bildausschnitt, bei dem die höhere Genauigkeit erzielt wurde, wurde in einem weiteren Schritt mit dem Conv3D-Modell mit den darauf zugeschnittenen Videos des Datensatzes A dann für den Vergleich der Farbräume RGB, Graustufen, HSV, LAB, XYZ und YcbCr verwendet. Die Genauigkeit wurde wieder in 5000 Trainingsepochen gemessen.

Die Modelle Conv3D, GRU und GRUConv wurden anschließend unter Verwendung des Bildausschnitts und Farbraums, welche zuvor die höchsten Genauigkeiten im jeweiligen Vergleich erzielten, mit dem Datensatz A trainiert und in 5000 Trainingsepochen anhand der Validierungsgenauigkeit verglichen.

Als finales Modell für das Lippenlesen wurde das neuronale Netzwerk mit der höchsten Validierungsgenauigkeit aus dem vorherigen Versuch von Grund auf, also ohne trainierte Parameter, mit dem Datensatz B (Tab. 1) trainiert. Der verwendete Zuschneidungsbereich und Farbraum vom vorherigen Versuch werden auch für das finale Modell übernommen. Das Modell wurde dann auf den Validierungsdatensatz B in jeder der 5000 Epochen angewandt, sodass die Parameter des Modells aus der Epoche mit der höchsten Validierungsgenauigkeit für das Modell übernommen werden. Die Genauigkeit des daraus resultierenden finalen Modells wurde anschließend mit dem Testdatensatz von Datensatz B sowie dem Datensatz C (Tab. 1) mit unbekannten Sprechern ermittelt. Die Genauigkeit des Lippenlesens wurde jeweils als Korrektklassifikationsrate gemessen und als Konfusionsmatrix dargestellt. Unter Verwendung der angegebenen Grafikkarten betrug die Trainingsdauer für alle Versuche insgesamt ca. 6 Wochen, wobei durchgängig ohne Unterbrechung trainiert wurde.

## Ergebnisse

### Modellvalidierung und Optimierung

**Figure 4:**
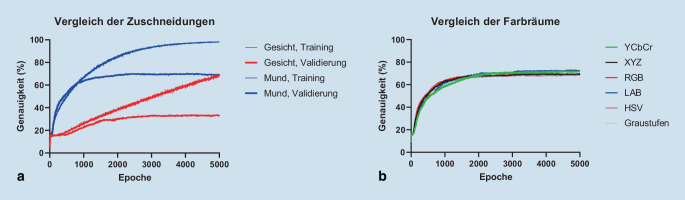

In Tab. 2 sind die Häufigkeiten der Videos pro Wortklasse zusammengefasst. Die Abb. 4a zeigt die Anwendung des Conv3D-Modells auf den für das Training verwendeten Teildatensatz A und den Validierungsdatensatz für 5000 konsekutive Epochen im Vergleich der Bildausschnitte. Auf den Trainingsdatensatz angewendet wurde bei Zuschneidung auf das Gesicht eine mittlere kontinuierliche, lineare Genauigkeitsverbesserung der Wortklassifizierung bis zu 70 % nach 5000 Epochen ermittelt. Bei Zuschneidung auf die Lippen erreichte das Modell eine Sättigung bei 99 % nach 5000 Epochen. Werden die Videos auf das Sprechergesicht zugeschnitten, erreichte das Modell in der Validierung eine Genauigkeit von 34 % nach 5000 Epochen. Bei Zuschneiden auf die Lippen wurde eine maximale Genauigkeit von 70 % bereits nach 2454 Epochen erreicht. Bei beiden Methoden des Zuschneidens wurde eine Überanpassung, d. h. eine Sättigung oder Verringerung der Validierungsgenauigkeit bei zunehmender Trainingsgenauigkeit, festgestellt.

**Table 2:** 

Wortklasse	Häufigkeit					
	Datensatz A		Datensatz B		Datensatz C	
	Anzahl	%	Anzahl	%	Anzahl	%
kommentare	118	3	387	1	72	2
fragen	171	5	918	3	154	4
prüfung	66	2	381	1	92	2
deutschland	56	2	422	1	138	3
können	325	9	2959	10	215	5
sprechen	131	4	846	3	182	5
wirklich	159	4	3225	11	174	4
eigentlich	194	5	1726	6	182	5
wissen	144	4	1110	4	65	2
natürlich	304	8	3212	10	323	8
video	308	8	1855	6	264	7
bedeutet	118	3	790	3	295	7
beispiel	585	16	3363	11	960	24
schreiben	106	3	932	3	158	4
menschen	274	7	2385	8	62	2
einfach	355	10	4435	14	360	9
wichtig	146	4	1501	5	118	3
wörter	167	4	237	1	136	3

Die Abb. 4b zeigt die Anwendung des Conv3D-Modells auf den für das Training verwendeten Teildatensatz A für 5000 konsekutive Epochen im Vergleich der Farbräume. Es ist ein nichtlineares Wachstum mit Sättigung nach ca. 2000 Epochen erkennbar. Es wurden maximale Validierungsgenauigkeiten von 70 % (2454 Epochen, RGB), 71 % (4970 Epochen, Graustufen), 69 % (4848 Epochen, HSV), 73 % (4684 Epochen, LAB), 72 % (4510 Epochen, YcbCr) und 70 % (3928 Epochen, XYZ) erreicht.

Im Vergleich der Modelle bei Verwendung des Mundbereichs als Bildausschnitt und des LAB-Farbraums wurden maximale Validierungsgenauigkeiten von 73 % (Conv3D, 4684 Epochen), 60 % (GRU, 2466 Epochen) und 78 % (GRUConv, 4297 Epochen) gemessen. Das GRUConv-Modell erreichte damit die größte Genauigkeit.

**Figure 5:**
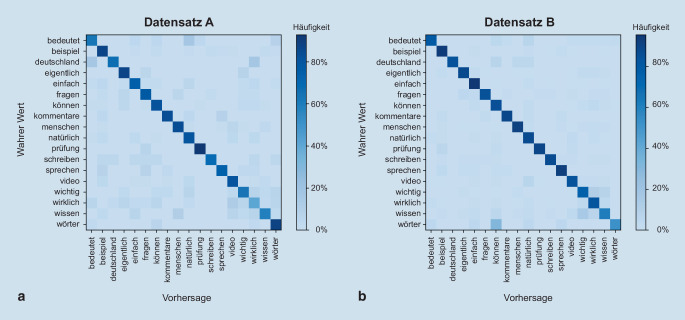

Bei Anwendung auf den Trainings- und Validierungsdatensatz B erreicht das GRUConv-Modell nach 1868 Trainingsepochen die maximale Genauigkeit von 87 % und damit eine im Vergleich zum Datensatz A verbesserte Performance. Auf den Testdatensatz von Datensatz B angewendet wurde eine Genauigkeit von 87 % erreicht. Abb. 5 zeigt die Konfusionsmatrizen für den Vergleich der vorhergesagten Wortklasse mit der wahren Wortklasse für die Datensätze A und B unter Verwendung des GRUConv-Modells. Die qualitative Auswertung der Matrizen zeigt sehr wenige Verwechslungen zwischen den Wortklassen und somit eine sehr große Sensitivität und Spezifität des Modells. Auf den Datensatz C mit unbekannten Sprechern angewendet erreichte das GRUConv-Modell eine Genauigkeit von 63 %.

## Diskussion

Diese Arbeit beschreibt einen Deep-Learning-Algorithmus, der bei einem definierten Datensatz an Videoaufzeichnungen Wörter anhand von Mundbewegungen mit hoher Treffergenauigkeit klassifiziert.

Ein wesentlicher Prozessschritt ist die Wahl des Bildausschnitts. Hier führte das Zuschneiden auf den Mund zu einer deutlich größeren Korrektklassifikationsrate als bei Zuschneiden auf das Gesicht. Ein Grund könnte sein, dass im Gesichtsbereich zu viele für das Klassifizieren unnötige Informationen enthalten sind, welche die Klassifizierung erschweren. Aufgrund einer komplexeren zu approximierenden Funktion ist für das Lippenlesen bei Verwendung des gesamten Gesichts als Bildausschnitt ein Modell mit anderen Hyperparameter, einem anderen Aufbau oder auch mehr Neuronen notwendig. Auch die verwendete Grafikauflösung des Gesichts von 90 × 90 Pixeln ist ein möglicher Grund für das schlechtere Abschneiden, da beim Skalieren relevante Informationen verloren gehen könnten. Eine höhere Auflösung wiederum würde die Rechenkapazität massiv erhöhen. Die Verwendung des Lippenbereichs hat sich auch in vergleichbaren Arbeiten im englischen Sprachraum durchgesetzt, so z. B. bei LipNet [2] und Google Deep Mind [29]. Andererseits ermöglicht auch das Verwenden von auf den Gesichtsbereich zugeschnittenen Videos, insbesondere unter Verwendung von Landmarkdetektoren [16], eine gute Performance. Diese kann sogar die Performances von State-of-the-Art-Modellen, die auf den Lippenbereich zuschneiden, übertreffen [9].

Bezüglich des verwendeten Farbraums zeigen sich kaum Unterschiede in der Korrektklassifikationsrate. Hierbei ist zu bedenken, dass Bilder des Mundbereichs ausgewertet wurden, bei denen per se eine geringe Vielfalt an Farben zu erwarten ist. Andererseits zeigen Gowda & Yuan [14], dass die verwendeten Modelle auch bei einem Datensatz aus unterschiedlichen Objekten für verschiedene Farbräume ähnlich gut performen. Auch dort konnte der LAB-Farbraum die höchste Genauigkeit erzielen und hatte im Vergleich zum zweitbesten Farbraum einen Genauigkeitsunterschied von weniger als 2 %. In der vorliegenden Arbeit ist nicht auszuschließen, dass bestimmte Wortklassen bei einigen Farbräumen treffsicherer klassifiziert werden. Möglicherweise ist dies der Grund, weshalb in dieser Arbeit im Graustufen-Farbraum mit vermeintlich weniger Informationen eine im Vergleich zum RGB-Farbraum höhere Genauigkeit erzielt wurde. Es ist nicht auszuschließen, dass gewisse Farbräume mit anderen Architekturen von neuronalen Netzwerken höhere Genauigkeiten erzielen würden. Zudem werden die Gewichte der Modelle zufällig initialisiert, sodass diese die Genauigkeiten ebenfalls beeinträchtigt haben könnten.

Bezüglich der untersuchten Deep-Learning-Modelle erreichte das GRUConv-Modell, welches CNN und GRU kombiniert, die höchste Korrektklassifikationsrate im Lippenlesen. Da die zugrunde liegenden GRU- und CNN-Schichttypen nicht verändert wurden, liegt es nahe, dass die Kombination der Modelle synergistisch wirken.

Das final verwendete Modell zeigt, dass Lippenlesen auf Deutsch mithilfe von neuronalen Netzwerken möglich ist. Die Genauigkeit von 78 % ist mit der für andere Sprachen erzielten Genauigkeit vergleichbar [15].

Ein großes Thema beim Lippenlesen sind Verwechslungen von Wörtern. Im verwendeten Testdatensatz waren die Erkennungsraten bei den Wortklassen „wichtig“, „wirklich“ sowie „wissen“ am niedrigsten und wurden im Vergleich zu den anderen Klassen öfter miteinander verwechselt. Dies ist zum einen damit zu begründen, dass die Mundbewegung ähnlich abläuft. Zum anderen war die Verteilung der Wortklassen über die Videos nicht homogen. Wiederum konnten für Klassen mit wenigen Videos, beispielsweise „prüfung“ und „deutschland“, relativ hohe Genauigkeiten gemessen werden. Diese Wörter haben sehr unterscheidbare und markante Mundbewegungen. Die Wortklassen „wichtig“, „wirklich“ und „wissen“ hingegen beinhalten relativ wenige für das Training zur Verfügung stehende Videos, aber ähnliche Lippenbewegungen. Die Frage, ob sich diese Wörter mit mehr Lernbeispielen besser unterscheiden lassen, wurde mit dem umfangreicheren Testdatensatz B beantwortet, bei dem sich größere Trefferraten zeigten. Auffällig ist hier nun, dass die Klasse „wörter“ oft mit „können“ verwechselt wird. Auch hier könnte die ähnliche Mundbewegung ursächlich sein.

Insgesamt muss diskutiert werden, dass, wie generell beim maschinellen Lernen, auch im Fall des Lippenlesens die Quantität an Trainingsdaten entscheidend ist. Wie in der Arbeit gezeigt wurde, ist der Umfang des Datensatzes relevanter für die Korrektklassifikationsrate als die Wahl des Modells.

Das final verwendete Modell hat eine gute Generalisierungsfähigkeit bei bekannten Sprechern gezeigt und wurde abschließend im Datensatz C getestet. Die hierbei verringerte Genauigkeit von 63 % war wegen der unbekannten Sprechenden zu erwarten, ist jedoch immer noch sehr hoch. Somit kann eine gewisse Generalisierung des Modells unabhängig von Sprechern angenommen werden.

Bei der Erzeugung der Datensätze könnten Fehler bei der Spracherkennung oder der automatischen Auswahl des relevanten Bildbereichs potenziell Einfluss auf das finale Ergebnis gehabt haben. Die Genauigkeit der automatischen Algorithmen in den manuell überprüften Testdatensätzen war jedoch sehr präzise, sodass dieser Einfluss als sehr klein eingeschätzt wird. Ein Vorteil des verwendeten Videomaterials ist, dass Videos des alltäglichen Sprachgebrauchs ausgewählt wurden und der Arbeit somit eine gewisse Relevanz für die Alltagskommunikation geben.

Insgesamt zeigt das finale Modell mit einer Genauigkeit von 87 % bei bekannten Sprechenden und 63 % bei unbekannten Sprechenden eine Möglichkeit des automatisierten Lippenlesens durch Deep Learning für die deutsche Sprache auf. Die Genauigkeit des Modells ließ sich mit einem erweiterten Datensatz verbessern. Die Klassifikation von mehr als 18 Klassen, also ein größeres Vokabular, ist eine weitere Verbesserungsmöglichkeit zukünftiger Arbeiten. Mit mehr Lernbeispielen sollte auch ein neuronales Netzwerk mit einem umfangreicheren und komplexeren Aufbau für das Lippenlesen anwendbar sein. Offen ist die weitere Suche nach optimalen Hyperparametern. Zukünftig könnte der entwickelte Algorithmus in einen Connectionist-Temporal-Classification-Algorithmus eingebettet werden um, von einem variabel langen Video den zugehörigen, gesprochenen Text zu ermitteln. Damit würde solch ein Modell keine Multiklassen-Klassifizierung wie in dieser Arbeit betreiben, sondern dem realen Lippenlesen nahekommen.

Das maschinelle Lippenlesen durch Deep Learning kann in vielen Feldern angewandt werden: Automatische Generierung von Untertiteln für Gehörlose und lautloses Diktieren in lauten Umgebungen sind ebenso potenzielle Anwendungsmöglichkeiten wie die Unterstützung von Spracherkennungssystemen.

Medizinisch kann der Algorithmus für Kehlkopfoperierte sehr nützlich sein, bei denen nach Anlage eine Tracheostomas die akustische Sprachproduktion meist nicht mehr möglich ist. Der Algorithmus kann in diesem Fall sehr schnell und mit hoher Genauigkeit auf die Lippenbewegungen der Betroffenen trainiert werden. Eine Einbettung in eine App könnte dann die Spracherkennung nahezu in Echtzeit möglich machen. Bei Anwendung auf beliebige Sprechende, wie es z. B. gehörlose Menschen benötigen würden, müsste das Modell generalisierbar sein. Diese Bedingung erfüllt das Modell ebenfalls mit einer akzeptabel hohen Trefferquote. In nächsten Schritt sollten Einbettungsmöglichkeiten in Smartphones und/oder Tablets entwickelt werden, damit möglichst viele Betroffene in der Alltagskommunikation davon profitieren.

## Schlussfolgerungen

In dieser Arbeit wurde erfolgreich ein neuronales Netzwerk zum Lippenlesen in deutscher Sprache umgesetzt. Es wurde ein Modell trainiert, dessen Genauigkeit die Eignung der Datensätze sowie die dabei eingesetzten Methoden bestätigt. Sowohl die Quelle der Daten als auch die dazu verwendete Sprach- und Munderkennung erwiesen sich als geeignet.
