# Erratum zu: Entwicklung und Evaluation eines Deep-Learning-Algorithmus für die Worterkennung aus Lippenbewegungen für die deutsche Sprache

**DOI:** 10.1007/s00106-022-01155-z

**Published:** 2022-03-15

**Authors:** Dinh Nam Pham, Torsten Rahne

**Affiliations:** 1 Universitätsklinik und Poliklinik für Hals‑, Nasen‑, Ohrenheilkunde, Kopf- und Halschirurgie, Universitätsklinikum Halle (Saale), Martin-Luther-Universität Halle-Wittenberg, Ernst-Grube-Str. 40, 06120 Halle (Saale), Deutschland; 2 Hochschule Anhalt, Köthen, Deutschland

**Erratum zu:**

**HNO 2021**

10.1007/s00106-021-01143-9

In diesem. Beitrag wurde versehentlich der Name des Autors **Dinh Nam Pham** fälschlicherweise als Nam Dinh Pham wiedergegeben.

Die Tab. 1 wurde ebenfalls nicht korrekt abgedruckt, bitte berücksichtigen Sie die u. a. Tabelle. Der Originalartikel wurde aktualisiert.

The original article has been corrected.

**Table 1:** 

Datensatz		Originalvideos	Sprechende	Videos im Datensatz
A	Gesamt	250	6	3727
	Training			2973
	Validierung			754
B	Gesamt	1400	22	30.684
	Training			24.553
	Validierung			3060
C	Test	156	4	3950
	Gesamt			3950

